# The Lords' Committee

**Published:** 1891-03-28

**Authors:** 


### The Lords' Committee.

The inquiry into the Brompton Hospital was resumed by the Lords' Committee on Thursday, the 19th inst., in order to hear Mrs. Walker's evidence as to the nursing arrangements. Mrs. Walker, who had been nine years Matron of the Brompton Consumption Hospital, stated that there were seventy-two nurses at Brompton, and they were very contented with their pay and food. During all the nine years she was there, she had never heard a single complaint from them, and if there had been any cause for complaint she would have been sure to have heard of it. The hospital nurses did not take part in the National Pension Fund. Some of the nurses were sent out to private patients, but it was certainly not the practice to take the nurses from the wards for that purpose. There were ward maids at the hospital, and they did the menial work; although, in the matter of polishing the floor, they were occasionally relieved for half-an-hour by the probationers. The Rev. Dr. Wace, Principal of King's College, and Chairman of the Committee of King's College Hospital, made a statement as to the income of the hospital, and said that they usually obtained a large sum from their annual festival. The last one realised about £2,500, but when the Prince of Wales presided at one of their dinners they obtained as much as £4,000. For want of funds some of the wards had to be closed about five years ago; but they were now all open, and even now so numerous were the applicants that many could not be admitted There were 220 available beds, and 215 were occupied last week. The system of drainage had been carefully gone into in 1882, and an entirely new system introduced. There was a plan of the drainage, and it was kept up to date. The number of in-patients treated last year was 2,528. There were 9,650 treated in the ordinary out-patient department, and 10,337 in the casualty department, and, in addition, 607 poor women had been attended to in their confinements. Those were all new cases. The total number of attendances was 29,556, excluding casualties. The nursing used to be conducted by the St. John's House Sisterhood, but six years ago there arose some difficulties from the dual control, and now the nursing was under a Sister-Matron employed by the hospital. All nurses must be members of the Church of England. In answer to a question from Lord Cathcart, Dr. Wace said they took subscriptions from persons of all religions, but he did not think it was unfair to have a religious test for the nurses, as it was well-known beforehand, nor had they found it to be an injudicious regulation. All hospitals have not such a rule, but he thought it was right that there should be some founded on that principle. Miss Monk, the Sister-Matron, then gave detailed evidence as to the hours of work, pay, food, and other matters connected with the nursing department. Dr. Curnow, F.R.C.P., dean of the medical school, followed, and explained the procedure adopted in admitting patients, and the relations of the school to the hospital and to King's College. As to special hospitals, he considered that, for the most part, they were unnecessary now that the general hospitals had established special departments. The Committee then adjourned until after Easter.

